# Case Report: Corneal Leucoma as a Novel Clinical Presentation of Nail-Patella Syndrome in a 5-Year-Old Girl

**DOI:** 10.3389/fped.2021.638630

**Published:** 2021-06-14

**Authors:** Ling Hou, Yue Du, Yubin Wu, Yue Zeng, Chengguang Zhao

**Affiliations:** Department of Pediatrics, Shengjing Hospital of China Medical University, Shenyang, China

**Keywords:** nail-patella syndrome, *LMX1B*, steroid-resistant nephrotic syndrome, congenital corneal leucoma, missense variation

## Abstract

Nail-patella syndrome (NPS) is a rare autosomal-dominant disorder characterized by the classic tetrad of absent or hypoplastic finger and toe nails, absent or hypoplastic patella, skeletal deformities involving the elbow joints, and iliac horns. This disease is caused by heterozygous pathogenic variations in the *LMX1B* gene, which encodes the LIM homeodomain transcription factor protein (LMX1B). We report a case of corneal leucoma and dysplasia prior to overt steroid-resistant nephrotic syndrome (SRNS) in a patient with NPS. At presentation, the parents of a 5-year-old female patient reported their daughter had corneal leucoma, psychomotor delay and speech defect. We also noted the presence of bilateral edema of the lower extremities, hypertension, nail dystrophy, and the bilateral absence of patella. She developed steroid-resistant nephrotic syndrome. Lowe oculocerebrorenal syndrome and NPS were the conditions considered in differential diagnosis. Trio-based whole genome sequencing indicated a heterozygous *de novo* likely pathogenic variation in the *LMX1B* gene (c.805A>C [p.Asn269His]). Patients with NPS often develop nail, ocular, or orthopedic symptoms prior to nephrotic syndrome. Corneal leucoma may be a novel clinical presentation of NPS.

## Introduction

Nail-patella syndrome (NPS, OMIM #161200) is a rare autosomal dominant disease that affects multiple developmental systems, including the integumentary and musculoskeletal systems, ocular system, neurologic system, and the kidneys. The classical clinical tetrad of NPS is nail dysplasia, elbow abnormalities, patellar aplasia/hypoplasia, and exostosis of the bilateral iliac wings (1). Kidney involvement, which typically manifests as proteinuria or even nephrotic syndrome, occurs in ~30 to 50% of cases and a small percentage of patients may progress to end-stage renal disease (ESRD) (2). The ocular anomalies of NPS include intra-ocular hypertension, glaucoma, and cloverleaf pigmentation of the inner margin of the iris (Lester's sign) (3, 4). Recent studies suggested that some patients may present with sensorineural deafness, short stature with hypothyroidism, schizophrenia, and internal carotid artery aplasia (5–8).

The incidence of NPS is ~1 per 50,000 live births. In 95% of cases with a clinical diagnosis of NPS, a pathogenic variation in *LMX1B* gene is detected. In 12.5% of cases the pathogenic variation occurs *de novo* in the affected patient (9–11). The *LMX1B* gene, located on the long arm of chromosome 9, codes for the LIM homeodomain (HD) transcription factor 1-beta protein (LMX1B; reference sequence: NM_002316.3). A normal *LMX1B* gene and its normal expression are essential for many developmental processes, such as dorsal-ventral polarization of the limbs, development of anterior eye structures, early morphogenesis of the glomerular basement membrane (GBM), and differentiation and migration of neurons in the central nervous system (1). Different heterozygous loss-of-function pathogenic variations in this gene account for the pleiotropic manifestations of the NPS phenotype.

In the present study, we describe a 5-year-old girl who presented with steroid resistant nephrotic syndrome (SRNS) and deterioration of renal function. We also noted limited ability of motion and remarkable intellectual disability. A physical examination indicated bilateral corneal leucoma, a smaller left eyeball, nail dystrophy, and bilateral edema of the lower extremities. Lower limbs X-ray detected the bilateral absence of the patellas. Whole genome sequencing analysis indicated a *de novo* missense likely pathogenic variation (c.805A>C [p.Asn269His]) within the homeobox of the *LMX1B* gene that was indicative of NPS. To our knowledge, only two previous reports identified NPS patients with pathogenic variations within the same amino acid code but different nucleotide patterns. Moreover, this is the first report of a patient with NPS and corneal leucoma.

## Case Report

A 5-year-old girl presented to our hospital with bilateral edema of the distal part of the lower extremities for about 10 days and hypertension for about 1 day. She was the second child of a non-consanguineous marriage and small for gestational age (SGA), with a full-term birth weight of 2.0 kg. At birth, her father was 53 years old and her mother was 51 years old. Her 32-year-old sister was apparently healthy. At birth a corneal opacity has been noted. Corneal leucoma has been diagnosed at the age of 7 months of life. At 1.5 years and 3.5 years, respectively, she undergoes to surgical interventions of cornea transplantation firstly in the right eye and then in the left one. After these operations, she was able to perceive light but still lacked vision. Her medical history was notable because of the presence of physical and intellectual disability. She likes to eat gruel, noodles and other easy to swallow food. She presented motor delay (she was able to sit and turnover, but not to climb, stand, or walk), cognitive and speech delay (she was able to tell only slow responses to questions, not able to spell). Moreover, her comprehension and memory were poor.

**Figure 1:**
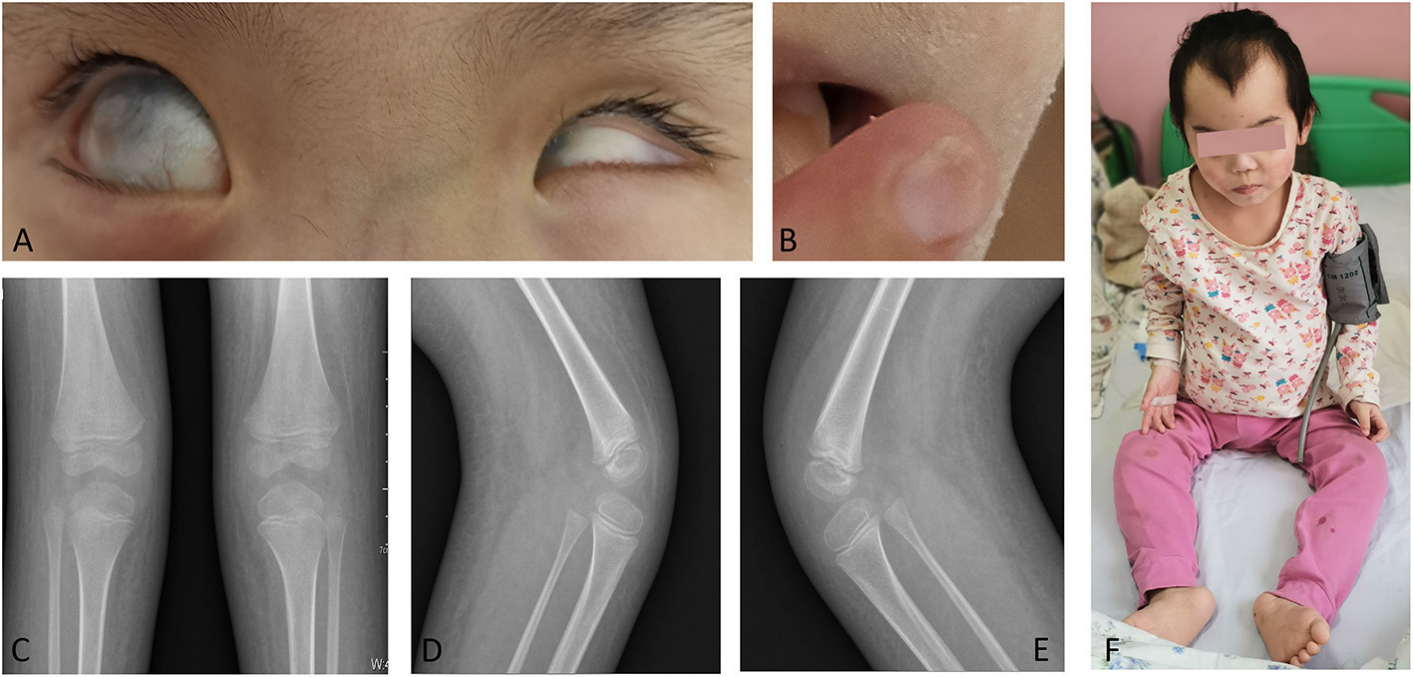
Major clinical features of the patient. **(A)** Corneal leucoma in both eyes, with no visible pupils and a small left eyeball. **(B)** Nail dystrophy (right index finger). **(C-E)** Bilateral absence of the patella. **(F)** The full body photo of the patient.

A physical examination indicated a temperature of 36.8°C, heart rate 112 times/min, respiratory rate of 25 times/min, blood pressure of 172/120 mmHg, stature of 95 cm (<3rd centile), head circumference of 49 cm (10th to 25th centile), unconsciousness, poor general condition, bilateral corneas completely covered with gray turbidity, no visible irises or pupils, a negative light reflex, a smaller left eyeball, an intraocular pressure of 21 mmHg in each eye. She had smooth breathing, nasal fan three concave negative, no skin rashes, no swollen superficial lymph nodes, without cyanosis, smooth oral mucosa, red pharynx isthmus, no blisters or ulcers, thoracic and symmetrical on both sides, symmetrical double lung auscultation breath sounds, strong and rhythmic heart sound, no murmurs, no abdominal tenderness, no touching of the liver and spleen under the ribs, pitting edema of the hands, legs and feet, capillary refill time <3 s, and nail dystrophy (Figure 1). Lower limbs X-ray detected the bilateral absence of the patellas (Figure 1). Laboratory data showed a large amount of protein in her urine with hypoalbuminemia, indicating nephrotic syndrome. Based on serological results, we excluded secondary causes of nephrotic syndrome, such as autoimmune disease or infection (Table 1). An ultrasound examination of her urinary system showed enlargement of both kidneys, especially the left kidney (Supplementary Figure 1). Echocardiography indicated a small amount of pericardial effusion (Supplementary Figure 2). Computed tomography of the head showed that her left eye was small with calcification (Supplementary Figure 3).

**Table 1:** Baseline laboratory characteristics of the patient.

**Variable**		**Reference range**
**Peripheral blood cells**		
White blood cells (×10^9^/L)	5.2	3.5–9.5
Red blood cells (×10^12^/L)	**3.7**	4–4.5
Hemoglobin (g/L)	**88**	120–140
Hematocrit (%)	**26.84**	37–47
Platelets (×10^9^/L)	180	135–350
**Blood chemistry**		
Total protein (g/L)	**30.6**	60–83
Albumin (g/L)	**12.6**	35–53
Aspartate aminotransferase (U/L)	17	5–34
Alanine aminotransferase (U/L)	6	0–40
Lactate dehydrogenase (U/L)	**331**	103–227
Urea nitrogen (mmol/L)	**3.96**	2.5–7.2
Creatinine (mmol/L)	35.5	45–84
Sodium (mmol/L)	135	135–145
Potassium (mmol/L)	**2.96**	3.5–5.5
Chlorine (mmol/L)	103.2	96–108
Triglycerides (mmol/L)	**2.47**	0.4–1.69
Total cholesterol (mmol/L)	**6.33**	3.36–5.69
C-reactive protein (mg/L)	**10.6**	0–8
Homocysteine (μmol/L)	7.83	0–15
Ceruloplasmin (g/L)	**0.169**	0.31–0.55
**Serology**		
Anti-nuclear antibodies	Negative	Negative
Rheumatoid factor	Negative	Negative
ANCA	Negative	Negative
Anti-GBM antibody	Negative	Negative
Immunoglobulin G (g/L)	1.17	4.81–12.2
Immunoglobulin A (g/L)	0.452	0.42–1.58
Immunoglobulin M (g/L))	**0.296**	0.41–1.65
Complement 3 (g/L)	**0.61**	0.74–1.4
Complement 4 (g/L)	0.134	0.12–0.36
Antistreptolysin O (IU/mL)	<25	0–200
Hepatitis B surface antigen	Negative	Negative
Anti-HCV antibody	Negative	Negative
**Urinalysis**		
Specific gravity	**1.031**	1.003–1.030
pH	6.50	4.5–8.0
Protein	**3+**	Negative
Red blood cells (per HPF)	**93.38**	0–3
Urinary protein (g/day)	**4.99**	0–0.15

*ANCA, anti-neutrophil cytoplasmic antibodies; GBM, glomerular basement membrane; HCV, hepatitis C virus; HPF, high power field*.

*The bold values stand for abnormal laboratory indicators*.

We initially administered oral corticosteroids as treatment for nephrotic syndrome. We also sequentially administered intravenous sodium nitroprusside and oral benidipine hydrochloride combined with losartan potassium to control blood pressure, which declined to ~130/85 mmHg. Although the edema was relieved, the persistence of severe proteinuria indicated corticosteroid resistance. A kidney biopsy was not performed because the parents did not provide consent.

**Figure 2:**
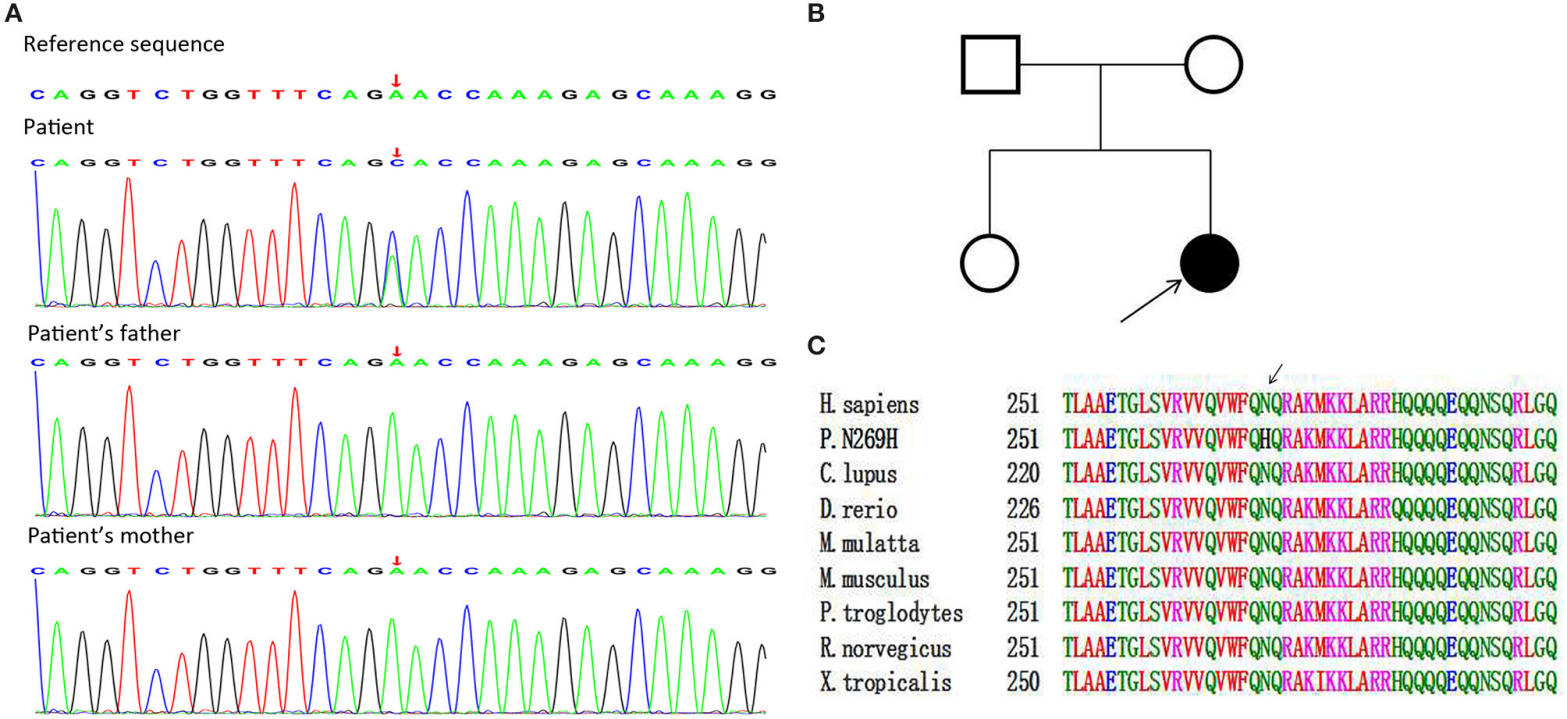
Genetic analysis. **(A)** Trio-based whole genome sequencing showing a heterozygous variation in the fifth exon of the *LMX1B* gene (805A>C [p.asn269his]) in the patient, but not her parents. **(B)** Patient pedigree. **(C)** Amino acid sequences of the LMX1B protein in different species, showing the variation was in the highly conserved homeodomain.

Oculocerebrorenal syndrome of Lowe and NPS were the main conditions to consider, given the clinical manifestations present in our patient, in the differential diagnosis. We therefore performed trio-based whole genome sequencing. In the patient the analysis showed the presence of a “*de novo*” heterozygous missense variation c.805A>C, corresponding to p.Asn269His (NM_002316, chromosomal location: chr9:129455866) in *LMX1B* gene, not described in any database. All bioinformatic analysis (SIFT, CADD, Polyphen2_HDIV, Polyphen2_HVAR, PROVEAN, MutationTaster, M-CAP, REVEL, GERP, phyloP20way, and phastCons20way software) predicted that this missense variation was likely pathogenic. This asparagine-to-histidine change was furthermore located in the HD domain, a region of the protein that is highly conserved among species and probably modulates DNA binding by the protein (Figure 2). Given the constellation of findings, we made a diagnosis of NPS likely caused by a *de novo* likely pathogenic variation in *LMX1B* gene. After this diagnosis, we rapidly tapered the oral corticosteroids. According to the clinical management of chronic kidney disease, we administered EPO and iron supplement for the anemia, antihypertensive agents to control blood pressure, and added calcitriol to maintain the balance of calcium and phosphorus. However, the family had economic difficulties and lacked compliance with these recommended treatments and follow-ups. At the 6-month follow-up, the patient still had significant proteinuria, hypoalbuminemia (serum albumin: 14.7 g/ L), an elevated serum creatinine (158.5 μmol/L), and severe anemia (hemoglobin: 50 g/L; peripheral blood erythroid cells: 2.1 × 10^9^/L), with occasional convulsions. Her parents refused further treatment and the patient was lost to follow-up.

## Discussion

We report the case of 5-year-old girl who presented with steroid-resistant nephrotic syndrome and had a medical history of corneal leucoma and multisystemic dysplasia (psychomotor delay and speech defect, nail dystrophy, and the bilateral absence of patella) since infancy. Based on the results of trio-based whole genome sequencing, we diagnosed NPS, a diagnosis that is compatible with many features of the patient (absence of the patella, nails abnormalities, renal disease, eye abnormalities). Ocular abnormalities are common in NPS, especially ocular hypertension (OHTN; prevalence of 7.2%) and open-angle glaucoma (OAG; prevalence of 9.6%) (3). The other reported ocular abnormalities in NPS are microcornea, sclerocornea, congenital cataracts, iris processes, and “Lester's sign” (4, 12, 13). The patient suffered from corneal leucoma and left microphthalmia, but had normal intraocular pressure. To the best of our knowledge, there are no prior reports of corneal leucoma in a patient with NPS.

The *LMX1B* gene has 8 exons and encodes a LIM-HD transcription factor which has an important role in development. The gene product of *LMX1B* has two LIM domains (LIM-A and LIM-B) and one HD. The LIM domains (encoded by exons 2 and 3) encode two zinc fingers that are essential in protein-protein interactions. The HD domain (encoded by exons 4 to 6) encodes a 60-amino acid region that is highly conserved among species and is necessary for DNA binding and regulation of transcription (1). The genome sequencing of our patient indicated a rare *de novo* variation (c.805A>C) in exon 5 of this gene. This new *LMX1B* variant implies the substitution of His with Asn at codon 269 in the HD domain of the protein. Although there are no reports of this missense variation in several databases (1000 Genomes Project, Leiden Open Variation Database, and ClinVar), previous studies reported that pathogenic variations within the same amino acid code but different nucleotide patterns (c.806-811del [p.Asn269-Gln270del]; c.807C>A [p.Asn269Lys]) caused NPS (14–16). Therefore, we hypothesize that the variant described here caused NPS in our patient due to haploinsufficiency of the transcriptional activity of *LMX1B*.

During embryonic development, LMX1B plays essential roles in regulating signal pathways that are responsible for establishing the normal dorso-ventral patterns of the limbs, kidney morphogenesis, and development of the eyes and central nervous system. Furthermore, LMX1B is ubiquitously expressed in the periocular mesenchyme and its derivatives, including the iris, ciliary body, and trabecular meshwork. Pressman et al. found that a homozygous pathogenic variation of *LMXIB* in mice led to iris and ciliary body hypoplasia, along with cornea stromal defects (13). Another study demonstrated that a functional LMX1B is necessary for development of the trabecular meshwork and maintaining corneal transparency in mice (12). Therefore, LMX1B appears to be important for the normal development of the cornea. There are no previous reports of NPS patients with corneal leucoma. It is possible that the corneal leucoma in our case was coincidental, rather than a specific manifestation of NPS. Therefore, further research is necessary to confirm the role of this rare *LMX1B* variant with corneal leucoma in NPS.

The prognosis of patients with NPS is determined by the severity of renal involvement, which can range from mild proteinuria to ESRD. NPS-associated nephropathy is characterized by irregular thickening of the GBM with electron-lucent areas (moth-eaten appearance) (17). During the development of mouse kidneys, LMX1B first appears in the S-shaped body region and then in the podocytes (9). This protein binds to the enhancer sequence of the type IV collagen α4 chain intron, thus regulating the expression of the type IV collagen α3 and α4 chains (18). Studies of conditional knockout mice showed that LMX1B is necessary for the normal differentiation and development of podocytes (19, 20). Moreover, LMX1B targets the *COL4A4, CD2AP*, and *NPHS2* genes and thereby affects formation of the slit diaphragm (20). However, podocin, CD2AP, and the type IV collagen α3 and α4 chains in podocyte specific *LMX1B* homozygous knockout mice persist after proteinuria, indicating these proteins apparently do not play an important role in the pathogenesis of the glomerular phenotypes of NPS (19). Slit diaphragm proteins are connected to the actin cytoskeleton, which has crucial importance for podocyte function. Time-resolved DNA microarray analysis after *LMX1B* inactivation in adult mice identified three genes encoding actin-associated proteins: transgelin, an actin-binding protein that stabilizes actin fibers; the actin-binding and Rho-activated protein ABRA, which promotes actin filament formation and/or stabilizes actin fibers; and a monomer GTPase ARL4c that may play a role in establishing focal contact. Burghardt et al. suggested that podocyte pathogenesis in NPS may be caused by dysregulation of the actin cytoskeleton (11, 21). A genotype-phenotype study suggested that pathogenic variations affecting the homeodomain of *LMX1B* affect the risk of developing nephropathy (22). The *LMX1B* variant in this study (c.805A>C [p.Asn269His]) is apparently extremely rare. A review of 41 families with NPS indicated the presence of the same p.Asn269Lys variation in two patients (23). One of these patients had nail, patella, and elbow hypoplasia, with nephropathy but no ocular involvement and the other patient had nail, patella, and elbow hypoplasia, with no renal or ocular involvement. Therefore, we hypothesize that the variant described here caused NPS in our patient. In addition, our patient also had remarkable physical and intellectual disability. Trio whole genome sequencing of patient and her parents showed no abnormality in genes associated with intellectual disability. LMX1B also functions in the central nervous system. Asbreuk et al. found that *Lmx1b* was detected in all brain areas, where late embryonic expression persisted, in restricted neuronal populations, suggesting functional cooperativity in the development of forebrain motor control systems (24). Doucet-Beaupré et al. found that *Lmx1a/b* are master regulator genes involved in the active maintenance of dopaminergic circuits throughout the lifespan (25). This may partially contribute to physical and intellectual disability.

In conclusion, we identified a *de novo* missense variation of *LMX1B* (c.805A>C [p.Asn269His]) as a probable pathogenic cause of NPS. This *LMX1B* variant was not previously reported in the literature. Our patient also presented with corneal leucoma, which was not previously described in a patient with NPS. Our NPS patient may thus have a novel clinical presentation due to this *LMX1B* genetic variant.

## Data Availability Statement

The original contributions presented in the study are included in the article/Supplementary Material, further inquiries can be directed to the corresponding author.

## Ethics Statement

The studies involving human participants were reviewed and approved by Ethics Committee of Shengjing Hospital of China Medical University (2018PS493K). Written informed consent to participate in this study was provided by the participants' legal guardian/next of kin.

## Author Contributions

LH reviewed the literature and contributed to manuscript writing. YD, YW, and YZ contributed to the acquisition and analysis of the clinical data. CZ was responsible for revision of the manuscript. All authors read and approved the final manuscript.

## Conflict of Interest

The authors declare that the research was conducted in the absence of any commercial or financial relationships that could be construed as a potential conflict of interest.
