# Supplementary material for: Case Report: Corneal Leucoma as a Novel Clinical Presentation of Nail-Patella Syndrome in a 5-Year-Old Girl
Source: Front Pediatr. 2021 Jun 14;9:638630. doi: 10.3389/fped.2021.638630 (PMC8236592; doi:10.3389/fped.2021.638630)
Supplement: Supplementary file 1 [file Data_Sheet_1.pdf]

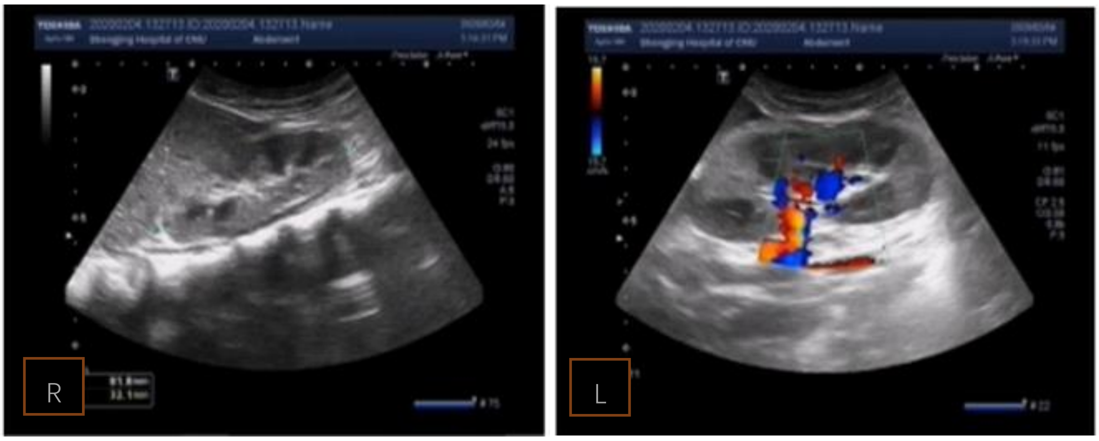

Supplementary Figure 1. Ultrasound of the urinary system indicating enlargement of the kidneys. The left kidney was 10.5×4.3×4.0 cm, the right kidney was 8.2×5.3×3.2 cm, and there was cortical echo enhancement and a blurred boundary between the cortex and medulla.

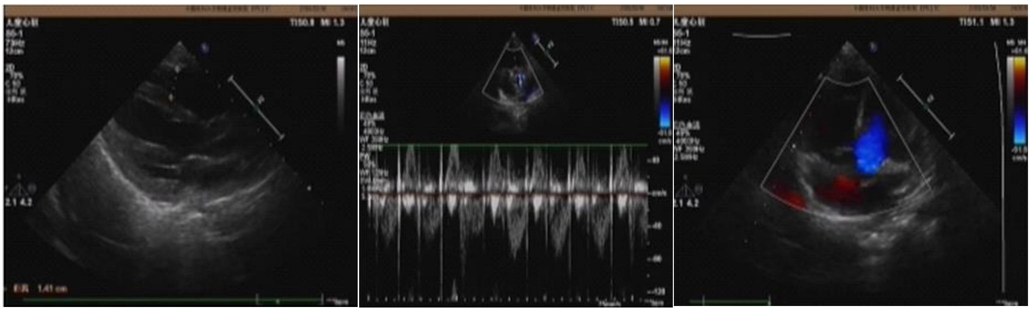

Supplementary Figure 2. Echocardiography showing a normal inner diameter of each cardiac cavity, integral atrioventricular septum, and functional valves. Trace reflux signals were present in the mitral and tricuspid valves, and a small amount of a pericardial effusion (about 3–5 mm) was also detected.

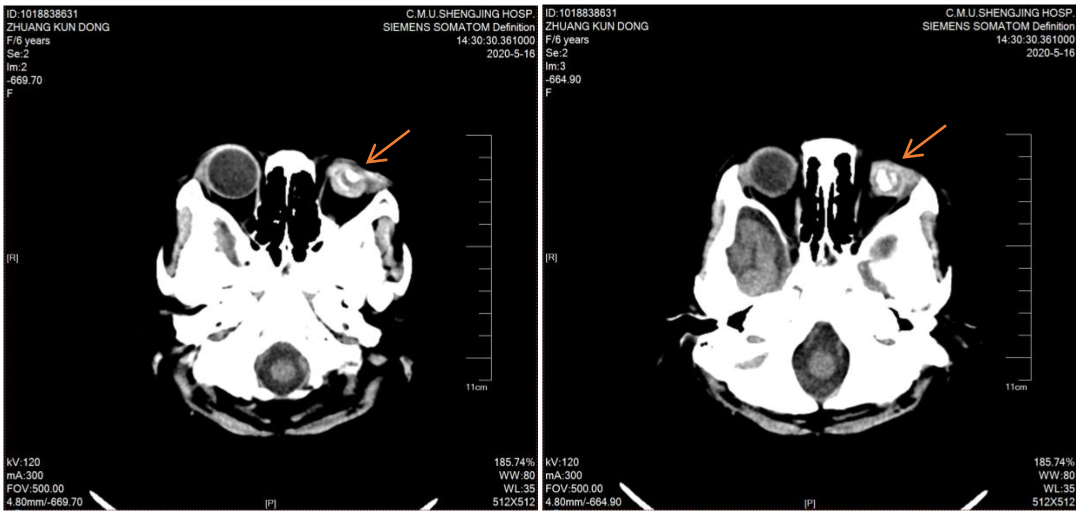

Supplementary Figure 3. Cranial computed tomography showing left eyeball atrophy and increased density with thick calcification (red arrow).
